# Refining Network Lifetime of Wireless Sensor Network Using Energy-Efficient Clustering and DRL-Based Sleep Scheduling

**DOI:** 10.3390/s20051540

**Published:** 2020-03-10

**Authors:** Ramadhani Sinde, Feroza Begum, Karoli Njau, Shubi Kaijage

**Affiliations:** 1 Department of Information Technology System Development and Management, Nelson-Mandela-AIST, Arusha 23311, Tanzania; 2 Faculty of Integrated Technologies, Universiti Brunei Darussalam, Gadong BE 1410, Brunei Darussalam; feroza.begum@ubd.edu.bn; 3 Department of Water Resources and Environmental Science and Engineering, Nelson-Mandela-AIST, Arusha 23311, Tanzania; karoli.njau@nm-aist.ac.tz; 4 Department of Communication Science and Engineering, Nelson-Mandela-AIST, Arusha 23311, Tanzania; shubi.kaijage@nm-aist.ac.tz or

**Keywords:** wireless sensor network, coronas, zone-based clustering, duty cycling, routing

## Abstract

Over the recent era, Wireless Sensor Network (WSN) has attracted much attention among industrialists and researchers owing to its contribution to numerous applications including military, environmental monitoring and so on. However, reducing the network delay and improving the network lifetime are always big issues in the domain of WSN. To resolve these downsides, we propose an Energy-Efficient Scheduling using the Deep Reinforcement Learning (DRL) (E^2^S-DRL) algorithm in WSN. E^2^S-DRL contributes three phases to prolong network lifetime and to reduce network delay that is: the clustering phase, duty-cycling phase and routing phase. E^2^S-DRL starts with the clustering phase where we reduce the energy consumption incurred during data aggregation. It is achieved through the Zone-based Clustering (ZbC) scheme. In the ZbC scheme, hybrid Particle Swarm Optimization (PSO) and Affinity Propagation (AP) algorithms are utilized. Duty cycling is adopted in the second phase by executing the DRL algorithm, from which, E^2^S-DRL reduces the energy consumption of individual sensor nodes effectually. The transmission delay is mitigated in the third (routing) phase using Ant Colony Optimization (ACO) and the Firefly Algorithm (FFA). Our work is modeled in Network Simulator 3.26 (NS3). The results are valuable in provisions of upcoming metrics including network lifetime, energy consumption, throughput and delay. From this evaluation, it is proved that our E^2^S-DRL reduces energy consumption, reduces delays by up to 40% and enhances throughput and network lifetime up to 35% compared to the existing cTDMA, DRA, LDC and iABC methods.

## 1. Introduction

Wireless Sensor Network (WSN) consists of various sensor nodes that are capable of sensing and communicating the data in the monitoring area. WSN proved to be an efficient requirement for the continuous monitoring of hostile areas such as environment monitoring like sudden volcanic eruptions, forest fires, floods and etc. [1,2,3]. The issues of reducing transmission delay and maximizing network lifetime are important research topics in the domain of WSN [4]. In order to reduce energy consumption and maximize network lifetime, two processes are included in WSN—clustering and duty cycling [5].

Cluster formation is one of the significant approaches in WSN to reduce energy consumption [6]. Clustering is a method of combining sensor nodes into clusters with a cluster head. Bio-inspired algorithms such as hybrid Particle Swarm Optimization (PSO) and Gravitational Search Algorithm (GSA) [7], Genetic Algorithm (GA) [8] and Multi-Objective Evolutionary Algorithm (MOEA) [9] are used to select the optimum cluster head in WSN. In hybrid PSO and GSA, the cost function is computed using subsequent parameters such as closeness to the cluster head and residual energy. The cost function is used to allocate the next hop for each cluster head to balance the load among cluster heads. In the HB algorithm, a set of cluster heads is selected from current nodes in the network and forms the clusters based on the cluster heads. In GA, a self-organizing network clustering method is proposed that affords a framework for dynamically optimized wireless sensor node clusters. In MOEA, an efficient cluster head is selected using two evolutionary algorithms such as GA and multi-objective PSO. Fuzzy-based cluster head selection is used [10,11,12] to select the best cluster head among sensor nodes in WSN. Here, the eligibility index is calculated for each sensor node for the election of the cluster head role.

Duty cycling is one of the significant methods to reduce energy consumption in WSN [13,14]. The Energy-aware scheduling with Quality Guarantee (ESQG) method is utilized [15] which aims at reducing sensor nodes’ energy consumption. The ESQG method dynamically adapts each sensor node awakening frequency. Each node dynamically turns on through node awakening probability and node importance. A distributed delay efficient data aggregation scheduling is used [16] for duty-cycled WSN. Here, the fast aggregation scheduling algorithm is used to schedule the sensor nodes. Delay aware tree construction and scheduling are proposed [17] for duty-cycled WSN. A Connected Dominated Set (CDS) tree is constructed for efficient scheduling. 

Routing is one of the significant processes to reduce delays during data transmission [18,19]. An energy-efficient scheduling-based cross-layer-based adaptive routing protocol is used [20] for WSN. The proposed routing protocol comprises of two models that include the network model and radio energy model. Energy-efficient clustering using multilevel routing is proposed [21] for WSN. The three different routing mechanisms are hierarchical routing using cluster identification, hierarchical routing using multi-hop and multi-level. A multi-objective hybrid routing algorithm is proposed [22] for WSN. In this, two approaches are used that are the scalarization approach and the lexico-graphical approach. The application of the WSN is evolving day by day owing to its wide range of amenities. However, the restricted power (battery source) of sensor nodes delegates the design of energy-efficient communication protocol for WSN [23].

In general, WSN has three routing protocols for communication, including a flat-based protocol, hierarchical-based protocol and location-based protocol. The flat routing protocols such as SPIN and directed diffusion have issues in scalability, overhead and latency [24]. This is due to the dissemination of information to all the nodes in the network. Besides, it also suffers from high link failures. The hierarchical routing protocols such as LEACH and PEGASIS have problems with the complexity in managing cluster head selection and routing the packets to the sink node [25]. Likewise, location-based routing protocols including GEAR, GAF and SPAN have high energy consumption, limited scalability and high overhead issues in WSN [26]. In addition, these protocols are not concentrated on the optimal cluster head selection while routing the packets. This increases energy consumption and reduces the stability of the WSN.

From the previously mentioned studies, we come to know that WSN still suffers from the issues that are discussed as follows:

- Energy consumption is still a major problem in the WSN. Most of the works concentrate on clustering to reduce energy consumption. However, it lacks a mechanism for the selection of optimal cluster head. This reduces data aggregation efficiency.
- The energy consumption of the sensor node is high due to the ineffective duty cycling process.
- Lack of parameter consideration in scheduling leads to frequent dead sensor nodes in the network.
- The network delay is high due to distance and energy-based path selection.

To resolve these shortcomings in existing communication protocols in WSN, our Energy-Efficient Scheduling using the Deep Reinforcement Learning (DRL) (E^2^S-DRL) approach constructs the following objectives:

- To reduce energy consumption during data aggregation for efficient cluster head selection and clustering in WSN.
- To schedule the state of the individual sensor node so as to reduce energy consumption.
- To find an optimal path to deliver the sensed data packet to the sink without routing overhead and delay.

The main aim of our work is to reduce energy consumption and network delay. The major contribution of this paper is summarized as follows:

- We locate our sensor nodes in the corona field to aggregate data from all sensor nodes effectually and also, we split coronas into zones to reduce energy consumption. To improve network lifetime, our work is based in three phases, namely Zone-based Clustering (ZbC), duty cycling and routing.
- The first phase reduces energy consumption during data aggregation through the ZbC scheme that comprises a hybrid PSO and the Affinity Propagation (AP) algorithm. PSO computes fitness function for following metrics energy factor and node degree. The energy factor is a combination of residual energy and distance between the source and sink node.
- The second phase enhances network lifetime through duty cycling. Duty cycling is performed using a DRL algorithm that schedules each sensor node in a distributed manner. Each scheduling slot considers three modes that are sleep, listen and transmit.
- In the last phase, routing is performed to reduce data transmission delay by finding the best path between source and cluster head. Ant Colony Optimization (ACO) algorithm is used to choose multiple paths between source and cluster head. Here, the ACO algorithm considers the following metrics to compute fitness functions such as residual energy, the distance between the source and the destination node, hop count and bandwidth. From the selected multiple paths, the best path between source and cluster head is selected using the firefly algorithm. The firefly algorithm considers succeeding metrics such as expected delay, packet delivery ratio and load.

The rest of this paper is structured as follows: Section 2 describes the state-of-the-art works related to the energy-efficient scheduling in WSN. Section 3 deliberates the proposed work with the algorithms discussed in detail. Section 4 illustrates the experimental results and comparative analysis. Section 5 discusses the research output. Finally, in Section 6 a conclusion and future work are briefly presented.

## 2. Related Works

This section provides a description of previous works related to energy-efficient scheduling in WSN. Here, we investigate three processes: clustering, duty cycling and routing. We have concentrated on these processes in our proposed work.

Palvinder et al. [27] have proposed optimal node clustering and scheduling in WSN. Herein, the improved Artificial Bee Colony (iABC) algorithm is used to select the optimal cluster head accompanied by optimal cluster head scheduling in WSN. The optimal cluster head selection is accomplished using four phases of the iABC algorithm. Here, the fitness function is computed using subsequent metrics, specifically: residual energy, minimum transmitted power to transmit aggregated data and distance between cluster head and the sink node. The compact BAT Bat algorithm is introduced by Trong et al. [28] in the WSN for clustering. It divides the WSN network into the unequal clusters. It selects the cluster head based on the consideration of energy consumed for transmitting and receiving the messages. The elected cluster head forms the clustering in the WSN network. It collects the data from the cluster member nodes to transfer into the sink node.

Islam et al. [29] have proposed cluster-based data aggregation in WSN. Here, sensor nodes are divided into several sensing areas using a clustering operation. The operation of distributed sensor nodes within each cluster is performed using the firefly algorithm. Cluster head selection operation is carried out using a combination of the firefly algorithm and the Low-Energy Adaptive Clustering Hierarchy (LEACH) model. The cluster-based firefly algorithm computes fitness function using distance and residual energy.

Jaeyoung et al. [30] have introduced the enhanced message-passing-based LEACH protocol for WSN. The enhanced LEACH (E-LEACH) protocol adapts the generalized energy consumption model while aggregating the data from the member nodes. It utilized the distributed algorithm to pass the message to the destination node. It selects the cluster head based on the remaining energy parameter. The elected cluster head participates in data aggregation and routing the data to the sink node.

The Energy-Efficient Duty Cycling (EEDC) algorithm is proposed in the wireless sensor nodes [31,32,33]. It balances both the delay and duty cycling of the sensor nodes. It allocates the slot for each node based on the energy consumed for the transmitting and receiving of the sensor node. It splits the duty cycling slots into three parts that are transmitting, receiving and listening modes. These slots are decided based on the energy consumed for each transmission and reception of the sensor network.

An adaptive wakeup interval-based duty cycling is presented by Adam et al. [34] in WSN. It adaptively changes the duty cycle of each sensor node based on the next wakeup interval time. It utilizes the adaptive function to select the next duty cycle of the sensor node. Here, the adaptive function performed based on the wakeup interval time of the sensor node.

Mohamed et al. [35] have proposed a cooperative scheduling protocol for sensor networks. The cooperative Time Division Multiple Access(cTDMA) scheduling was used to schedule the sensor nodes. Here, each sensor node follows the duty cycle to determine how often it will become Cluster Head (CH) in each round, that was decided at the beginning of each round. The cooperative TDMA (cTDMA) slot was divided into a direct transmission sub-slot and cooperative transmission sub-slot. During the direct transmission sub-slot, the active node transmits its packets to the sink and cooperative nodes. The relay nodes transmit their packets in a cooperative transmission sub-slot. The proposed cTDMA cannot support the dynamic changing of slots in scheduling.

Ngoc et al. [36] have proposed an efficient minimum latency scheduling algorithm for WSN. Here, Independent Set based Scheduling (ISS) algorithm is used. An independent set-based scheduling algorithm aggregates a fixed number of data into one packet to reduce the data transmission delay. To minimize the required time slot for sensor nodes, this method forwards as many packets from the source node to the destination node. The proposed method has less sleep time that leads to high energy consumption.

Mohammed et al. [37] have proposed an enhancement approach for reducing energy consumption in WSN. The cluster head is selected based on the number of neighbors count and residual energy of the node. Herein, the highest neighbor count node acquires more chances to select as a cluster head. A modified TDMA algorithm is used for scheduling that contains two phases: setup and steady. The steady phase has more time slots than the setup phase. The largest cluster has minimal sleep time compared to the smallest cluster. The largest cluster has more active time that leads to a decrease in the network lifetime.

Supreet et al. [38] have proposed hybrid meta heuristic-based routing for WSN. A hybrid ACO and PSO algorithm based energy-efficient protocol are used to form a cluster. An ACO-based path selection technique is utilized that forms a spanning tree between the cluster head and sink node. Here, the next hop is selected based on the distance between nodes.

The heuristic algorithm ACO-based self-organized energy-balanced algorithm is utilized in WSN [39,40]. The proposed method consists of three phases: cluster formation, multipath creation and data transmission. In cluster formation, the desired numbers of sensor nodes are selected as a cluster head and the remaining nodes join the nearest CH to form the cluster. Multiple paths between cluster head and cluster member nodes are explored using the ACO algorithm. ACO selects an energy-efficient optimized route for data transmission between cluster members and cluster head nodes. The above discussed routing algorithm has a slow convergence rate, and therefore, the next-hop selection is not effective.

Hana et al. [41] have proposed a Multi-Hop Graph-based approach for Energy-Efficient Routing (MH-GEER) protocol for WSN. MH-GEER deals with node clustering and inter-cluster multi-hop routing selection phase. In the clustering phase, the K-Means algorithm is utilized to form centralized clusters based on the randomly set ‘K’ clusters. In the routing phase, a new inter-cluster routing protocol is used to find a path between the cluster head and sink node. Here, a cluster head launches an agent to carry data and find a path to the sink node to deliver the carried data.

Feng et al. [42] have proposed a distributed routing algorithm for WSN. The distributed routing strategy is utilized to achieve better data aggregation for WSN. The proposed algorithm achieves better trade-off between latency and energy conservation. This method finds the global-optimal path to achieve a minimum average end-to-end delay with less energy consumption. The above-discussed routing methods have more data transmission delay, since this process does not consider the distance between the sink node and cluster head, which leads to more energy consumption. 

Jiang et al. [43] have proposed a Low Duty-Cycle (LDC) mechanism to reduce the latency and energy consumption in WSN. Here, nodes are woken up based on the neighbor’s wakeup time which leads to the increase in energy dissipation of nodes. 

Vijayalakshmi and Manickam [44] have proposed the Space Division Multiple Access (SDMA) mechanism to gather data from the WSN nodes. In this, CH selection was not effective due to the absence of significant parameter consideration such as distance and energy.

Table 1 illustrates the related work description with its demerits. Here, the existing methods with its contribution, objective and demerits are discussed in detail.

## 3. Proposed Work

**Figure 1:**
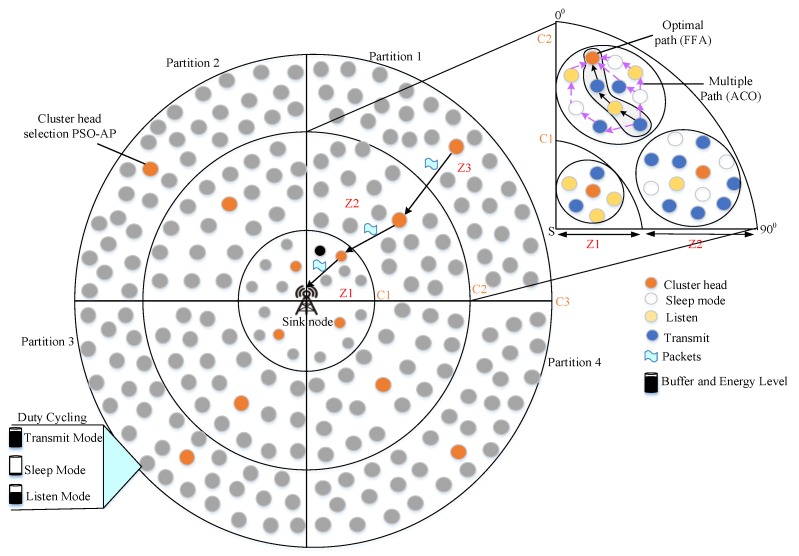
The architecture of proposed work.

Our proposed work enhances network lifetime through energy-efficient scheduling using the DRL algorithm in WSN. Our network comprises static sensor nodes and sink nodes as depicted in Figure 1. We consider our sensing field as three coronas in which sensor nodes are deployed. A sink node is deployed in the center point of the coronas. Each corona is split into four partitions based on the sink node position. Our work is composed of three sequential phases, namely, ZbC, duty cycling and routing. The first phase diminishes energy consumption through data aggregation using the ZbC scheme. In the ZbC scheme, a hybrid PSO and AP algorithm are used to establish clusters in each zone. Herein, PSO is applied to choose the best exemplar for AP using fitness function computation which contemplates succeeding metrics such as node degree, residual energy and distance. Based on the elected exemplar, AP forms clusters in each zone efficiently. The second phase enhances network lifetime through duty scheduling that is accomplished using the DRL algorithm. The proposed scheduling algorithm adaptively changes each sensor node scheduling mode. In the last phase, network delay is minimized via the ACO and FFA algorithms. Here, FFA is used to select the best path from multiple paths selected by ACO.

ACO selects multiple paths by considering subsequent parameters that are residual energy, hop count, bandwidth and distance. Firefly Algorithm (FFA) selects the best path by taking into account the following metrics, for instance, expected delay, packet delivery ratio and load.

### 3.1. Clustering Phase

In WSN, clustering plays a vital role in terms of energy consumption since it minimizes energy consumption during data aggregation. To avoid this problem, we pursue the ZbC scheme that forms an efficient cluster in each zone using hybrid PSO and AP algorithms. The shortcoming of the AP algorithm is that the number of exemplar selections is not effective. To avoid this problem, we combined PSO with AP. The reason for selecting the PSO algorithm is that it has a high probability and efficacy in identifying the global optimal solution. It pursues the intelligence behavior of swarms that provides effective searching results. It is necessary for our proposed AP cluster formation in order to enhance the network lifetime effectually and also reduces the frequent cluster head selection due to earlier death.

#### 3.1.1. PSO based Exemplar Selection

The PSO algorithm operates based on the swarming nature of flocks of birds. In our work, we use the PSO to choose an optimum exemplar for the AP algorithm. The PSO computes the fitness function for subsequent metrics, including node degree, residual energy and distance. The PSO selects an exemplar in each zone to form effective clusters. Here, the node that has the highest fitness function is elected as the exemplar for the AP-based clustering process. The PSO comprises three phases that are (i) initialization, (ii)fitness function evaluation (iii) global best ($G_{b}$) and local best ($L_{b}$) computation. These processes are discussed as follows:

(i) Initialization

In PSO, each sensor node is considered as a particle that is associated with an initial position, node degree, residual energy and distance.

(ii) Fitness function computation

In this phase, PSO computes a fitness function for each node by means of succeeding parameters such as node degree, residual energy and distance that are explained as follows:

(I) Node degree

Node degree is described as the number of neighbor nodes connected to the particular node. This metric selects the node that has the highest node degree that can communicate with more neighbor nodes that tends to increase in network performance. Node degree is represented as ‘$N_{D}$’ that can be conveyed as follows:

(1) $$N_{D}=\;N_{c}\left(N_{i}\right)$$

where, $N_{c}\left(N_{i}\right)$ represents the neighbor count of the node $N_{i}$.

(II) Residual Energy

Residual energy is described as the difference between total primary energy and consumed energy. Herein, the residual energy metric is used to select the highest energy node to avoid the frequent death of sensor nodes in the network. It is represented as ‘$E_{r}$’ and is expressed as follows:

(2) $$E_{r}=\;E_{p}-\;E_{c}$$

where, $E_{p}$ represents total primary energy and $E_{c}$ represents consumed energy.

(III) Distance

The distance parameter is referred to as the distance between the sensor and the sink node. This metric is also involved in network delay, since the distance is directly proportional to the delay. It is represented as ‘$D_{i,s}$’ and is expressed as follows:

(3) $$D_{i,s}=\;\sqrt{\left({\left(N_{s,x}-N_{i,x}\right)}^{2}+{\left(N_{s,y}-N_{i,y}\right)}^{2}\right)}$$

where,$\;N_{s,x},\;N_{s,y}$ represents the position of the sink node and $\left(N_{i,x},N_{i,y}\right)$ represents the position of the sensor node. By means of the above metrics, PSO computes the fitness function for the exemplar using the following expression [42],

(4) $$f_{e}=\;\sigma_{1}\left(E_{r}\right)+\;\sigma_{2\;}\left(D_{i,s}\right)+\;\sigma_{3}\left(N_{D}\right)$$

where,$\;\sigma_{1}$, $\sigma_{2\;}$ and $\sigma_{3}$ are weightage parameters.

(iii) Global Best and Local Best Computation

After computing the fitness function for each node, this phase compares each node local best $‘L_{b}’$ with another node to find the global best $‘G_{b}’$ node. This process is continued until the stopping criterion reached. Finally, the optimum node with the highest fitness value is elected as an exemplar for the AP process.

#### 3.1.2. AP Cluster Formation

Based on selected exemplar AP forms clusters, here exemplar nodes are elected by computing the fitness function for each node. Using selected ‘k’ exemplar, AP forms clusters by means of similarity between sensor node $N_{i}$ and exemplar $N_{e}$ that is computed using the following expression,

(5) $$Sim\;\left(N_{e},\;N_{i}\right)=\;{\left|N_{e}-N_{i}\right|}^{2}$$

where ${\left|N_{e}-N_{i}\right|}^{2}$ represent the similarity between $N_{i}$ and $N_{e}$ in terms of Euclidean distance. AP has two matrices that are the responsibility matrix and available matrix. The responsibility matrix and available matrix are updated using a computed similarity. Responsibility matrix ‘$R_{m}$’ is updated as follows:

(6) $$R_{m}\left(N_{e},\;N_{i}\right)=Sim\;\left(N_{e},\;N_{i}\right)-max_{i^{\prime }\neq i}\;(A\left(e,i^{\prime }\right)+s\left(e,i^{\prime }\right))$$

where, $A\left(e,i^{\prime }\right)$ represents the availability matrix that is expressed as follows:

(7) $$A\left(e,i^{\prime }\right)=\min\left(0,\;R_{m}\left(N_{e},\;N_{i}\right)\right)+\sum_{i^{\prime }\neq e,i}\max\left(0,R_{m}\left(N_{e},\;N_{i}\right)\right)$$

Using the above two equations, the responsibility matrix and the availability matrix is computed for each sensor node and exemplar node. This process is repeated until possible clusters are formed. Our proposed clustering hybrid algorithm forms effective clusters in each zone that reduces energy consumption through data aggregation.

### 3.2. Duty Cycling Phase

Duty cycling is one of the significant processes to reduce the energy consumption of WSN. In our work, we proposed the DRL algorithm for duty cycling that adaptively decides each node’s scheduling mode.

#### DRL-Based Scheduling

The DRL algorithm is utilized to schedule each sensor node’s scheduling modes adaptively. In our work, we divide the time period into the different slots that are allocated to each sensor node. Hence, each sensor node contains a unique slot that tends to avoid data collision between sensor nodes during transmission. In each slot, nodes adaptively change their mode using the DRL algorithm. In our work, we consider three modes in scheduling, which are sleep, listen and transmit that are conveyed as follows:

(a) Sleep Mode

During sleep mode, each sensor node turns off its radio that reduces the energy consumption of each node which in turn increases the lifetime of the network. A sensor node cannot receive or transmit any data when it is sleeping.

(b) Listen Mode

In listen mode, each sensor node senses their surrounding field and also receives data from neighbor nodes. During listen mode, a sensor node turns off its transmitter circuitry, hence the transceiver can receive data only.

(c) Transmit Mode

In transmit mode, each sensor node transmits its sensed data to the sink node and also it transmits data from the neighboring sensor node.

**Figure 2:**
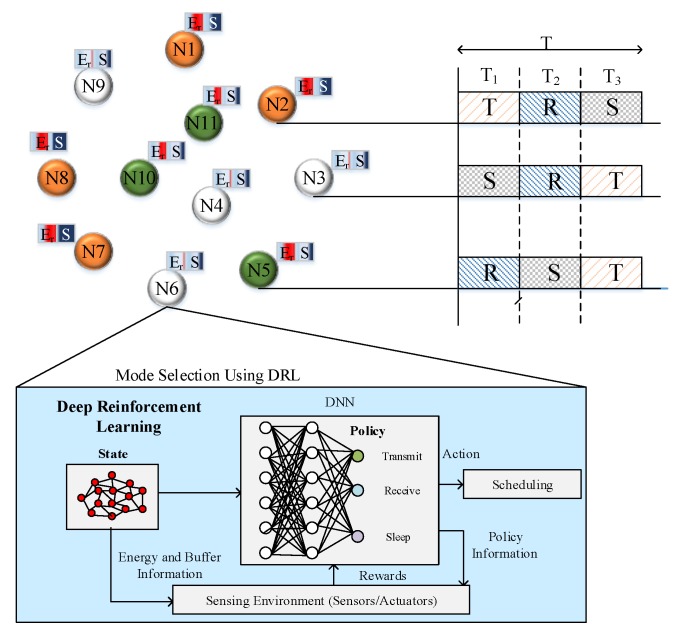
Deep Reinforcement Learning (DRL) scheduling.

Figure 2 depicts the DRL scheduling of the sensor nodes with three modes such as Transmit (T), Sleep (S) and Receive (R). Each node changes these three modes in each period using the DRL algorithm. In DRL, we adopt deep Q-Learning that comprises of following operations such as actions, states, Q-value, payoff and reward. A deep Q-leaning algorithm learns the environment state information and obtains the best action. The steps involved in DRL scheduling are discussed as follows:

(i) Actions (A): In our work, we propose three actions that are Sleep (1), Listen (2) and Transmit (3). Actions are undertaken by each node based on the computed Q-value.
(ii) States: We propose four states for each node that are $S_{0},\;S_{1},\;S_{2}\;$ and $\;S_{3}$. $S_{0}$ indicates the node does not have any storage in its buffer BS=0. $S_{1}$ indicates a node has storage in its buffer as $\frac{BS}{BS}$. $S_{2}$ indicates a node has storage in its buffer as $\frac{BS}{4}$. $S_{3}$ indicates a node has storage in its buffer as $\frac{BS}{2}$.
(iii) Q-value: Q-value is computed to select actions for each node. Here, Q-value is computed using the payoff value of the particular node.
(iv) Payoff: Payoff value is computed using the probability of the node to select an action. The payoff value is computed for each node to select particular action in each period.
(v) Reward: Reward is provided to each action on the basis of the successful transmission of packets to the neighbors.

Consider two nodes $N_{i}\;$ and $N_{j}$ need to select actions during the allocated period then it determines the payoff value for each node. This payoff value is computed using probability distribution function over actions. If the node $N_{i}$ and $N_{j}$ selects action l and m respectively, then node $N_{i}$ receives payoff $N_{i_{l.m}}$ and node $N_{j}$ obtains payoff $\;N_{j_{l,m}}$. Let $\alpha_{1}-\alpha_{3}$, denote probability for node $\;‘N_{i}$’ to select actions 1 to 3 where $\;\alpha_{1}+\alpha_{2}+\alpha_{3}=1$. Let $\beta_{1}$ - $\beta_{3}$ denote probability for node $\;‘N_{j}$’ to select actions 1 to 3 where $\;\beta_{1}+\beta_{2}+\beta_{3}=1$. The node $‘N_{i}’$ expected payoff is represented as:

(8) $$P_{Ni}\;=\;\sum_{1\leq l\leq 3}(\sum_{1\leq m\leq 3}N_{i_{l,m}}\alpha_{l}\beta_{l})$$

The node $N_{j}$ expected payoff is represented as,

(9) $$P_{Nj}\;=\;\sum_{1\leq l\leq 3}(\sum_{1\leq m\leq 3}N_{j_{l,m}}\alpha_{m}\beta_{m})$$

where payoff values $N_{i_{l.m}}$ and $N_{j_{l.m}}$ for nodes $N_{i}\;$ and $N_{j}$ are defined as the energy utilized by each node. This energy represents the remaining energy of the node which is calculated using Equation (2). The reward is given to each node if the packet is successfully transmitted that is represented as  ‘ℜ’. This factor is included in energy consumption only if the packet is successfully transmitted.

For example, if node $‘N_{i}’$ has packets to transmit, selects transmit action, then node $‘N_{j}’$ selects the listen action that induces packets that are successfully transmitted. Here, payoffs for both nodes are positive, which can be calculated using energy consumed to transmit/receive packet plus reward constant  ℜ. In the transmit state node $‘N_{i}^{’}$ intends to transmit a packet that consumes energy $\sigma_{ei}$ and $N_{j}$ consumes energy $\sigma_{ej}$ to receive packets. Then, the payoff for node $N_{i}\;$ is $-\sigma_{ei}+ℜ=P_{N_{i}}$ and the payoff for node $N_{j}$ is $-\sigma_{ej}+ℜ=P_{N_{j}}$. This way of packet transmission reduces collision effectually.

The Q-value is computed using the following equation,

(10) $$Q\left(S,A;\theta \right)=\;E\left({ℜ}_{i},+\;\delta {ℜ}_{2}+\;\delta {\;}^{2}\;{ℜ}_{3}+\ldots \right)$$

where δ represents the discount rate for action and θ is weight parameter. The discount rate determines the importance of future rewards. The value of the discount rate relies on the range of [0 to 1]. Thus, δ=0, represents that a node is biased in current rewards whereas δ=1 represents that a node achieves a high reward. The deep Q-learning learns the parameter θ of the action function Q(S,A;θ) by minimizing sequence of the loss function, where *i*th loss function $L_{i}\left(\theta_{i}\right)$ is given by,

(11) $$L_{i}\left(\theta_{i}\right)=\;E\left[{ℜ}_{n}+\;\begin{array}{c} max\\ A_{n+1} \end{array}\;Q\left(S_{n+1},A_{n+1};\theta_{i-1}\right)-Q\left(S_{n},A_{n};\theta_{i}\right)\right]$$

where, $\theta_{i}$ is the neural network parameter at *i*th update and parameters from the previous update $\theta_{i-1}$ are held to be fixed during the optimizing of the loss function $\;L_{i}\left(\theta_{i}\right)$. The term ${ℜ}_{n}+\;\begin{array}{c} max\\ A_{n+1} \end{array}\;Q\left(S_{n+1},A_{n+1};\theta_{i-1}\right)-Q\left(S_{n},A_{n};\theta_{i}\right)$ is the target for iteration i that depends on the neural network parameters from the last update. Differentiating the loss function with respect to the neural network parameter at iteration i, $\theta_{i}$ gives the upcoming gradient,

(12) $$\nabla \theta_{i}L_{i}\left(\theta_{i}\right)=E\left[{ℜ}_{n}+\;\begin{array}{c} max\\ A_{n+1} \end{array}\;Q\left(S_{n+1},A_{n+1};\theta_{i-1}\right)-\;Q\left(S_{n},A_{n};\theta_{i}\right)\nabla \theta_{i}Q\left(S_{n},A_{n};\theta_{i}\right)\right]$$

where, $\nabla \theta_{i}$ represents gradient of $\theta_{i}.$
**Algorithm 1. Node Scheduling****Require:** Energy and Buffer of Node **Ensure:** Selected Mode of Operation for Node  Initialize → replay memory D to the capacity ‘C’ *Initialize* → Q network with random weights θ. *Initialize* → target Q network with random weights$\;\theta^{-}=\theta$ **for**
$\left(S_{i}<S\;\&\&\;A_{i}<A\right)$**do**
 {  payoff 𝒫 select → action A;  payoff 1-𝒫 select →
$A_{n}$ maximizes S,A;θ;  }   *Execute* → action $A_{n}$ to obtain reward ${ℜ}_{n}$ and $S_{n+1}$
 *Store* ($A_{n},\;S_{n},\;S_{n+1},\;\;{ℜ}_{n}$) →
D **If** (n==n+1)  Set target →
${ℜ}_{n}$; **Else**
 Set target →
${ℜ}_{n}+\;\begin{array}{c} max\\ A_{n+1} \end{array}\;Q\left(S_{n+1},A_{n+1};\theta^{-}\right)$;  *Update*
$\theta^{-}\to \theta$; **End for**


Algorithm 1 describes how the proposed work that selects the mode of operation (Transmit (T), Sleep (S) and Receive (R).) for each node in each time slot. Initially, reply memory  D, weight θ  and random weight parameters $\theta^{-}\;$ are initialized. At first, the node selects an action that depends on the probability distribution over three actions sleep, listen and transmit respectively in the current state. A node carries out the selected action and observes the reward and new state. The node adjusts the probability distribution over three actions in the state S based on the payoff. In each iteration, $\theta^{-}$ the target parameter is updated effectually. In this way, we schedule each sensor node in the network that tends to reduce collision and also reduces the energy consumption of the sensor node.

In order to avoid divergence of the DRL, two techniques are introduced that are experience replay and the fixed target network. Using these techniques, DRL minimizes the loss function,

(13) $$L_{i}\left(\theta_{i}\right)=\;E_{S_{n,},A_{n,}{ℜ}_{n},S_{n+1}}\sim D\left[{ℜ}_{n}+\;\begin{array}{c} max\\ A_{n+1} \end{array}\;Q\left(S_{n+1},A_{n+1};\theta {\;}^{-}\right)-Q\left(S_{n},A_{n};\theta_{i}\right)\right]$$

where D  denotes experience replay memory and $\theta {\;}^{–}$ are the parameters of the target Q-network. Using these equations, a deep Q-learning algorithm adaptively changes each node’s action effectually.

### 3.3. Routing Phase

Transmission delay is more in WSN due to inefficient path selection between the source and the sink node. To address the transmission delay problem, the ACO and FFA algorithm-based routing is proposed. Multipath selection reduces delay in data transmission between source cluster member (CM) and exemplar. Hence, we first select multiple paths between CM and exemplar using ACO. From the selected multipath, an optimum path is selected using FFA. The description of ACO and FFA algorithms can be discussed as follows:

#### 3.3.1. ACO Algorithm

The proposed ACO algorithm selects the multiple paths between the source and destination nodes. The reason behind selecting this algorithm for multipath selection is that it provides a better searching behavior among the population in parallel. It provides an optimal solution rapidly by using the effective pheromone function. This pheromone function has benefits in the selection of multiple paths between the source and destination. The ACO algorithm follows the ants’ behavior which has the habit of pheromone evaporation during moving. Thus, it increases the probability of finding an optimal path during moving. With the aid of this pheromone behavior, the ACO algorithm estimates the pheromone function that tends to yield an effective solution in path estimation. Hence, we selected this algorithm for multiple path selection between source and destination. The ACO algorithm works on the basis of real ant behavior. The ACO algorithm computes the fitness function using subsequent parameters such as hop count, bandwidth, residual energy and distance. In ACO, the multipath is selected based on the pheromone value of each node. Pheromone value is updated in each iteration to discover the best path between the source CM and the exemplar. Pheromone value is computed using the following parameters that are explained as follows:

(i) Hop Count

Hop count is described as the number of nodes between source CM and exemplar. This metric is used to reduce delays in data transmission since delays tend to increase the energy consumption of the sensor node. It is represented as ‘$H_{c}$’ that is explained as follows:

(14) $$\;H_{c}=N_{c}\left(N_{CM_{i}},N_{e}\right)$$

where, $N_{c}\left(N_{CM_{i}},N_{s}\right)$ represent the hop count between source cluster member node $N_{CM_{i}}$ and exemplar $\;N_{e}$.

(ii) Distance

The distance parameter is referred to as the distance between the source cluster member and the exemplar node. This metric also involved in network delay, since the distance is directly proportional to the delay. It is represented as ‘$D_{CM_{i,e}}$’ that can be computed as same as Equation (3).

(iii) Bandwidth

The bandwidth parameter is considered in order to know the capacity of the link. Typically, bandwidth is measured in terms of bits per second. The bandwidth metric is used to select the best path to route the sensed data. It is represented as ‘$B_{p}$’ that is expressed as follows:

(15) $$B_{p}=\frac{b_{n}}{s}$$

where, $b_{n}$ indicates the number of bits and s indicates seconds.

In proposed ACO algorithm ants are working based on two rules. They are named as the initial rule and revising rule. These rules are briefly explained as follows:

(a) Initial Rule

In this rule probability of selecting a path is computed using the pheromone. The pheromone function is computed using fitness value and pheromone intensity. At first, ACO computes fitness values for each node to elect multiple paths between the source CM and the exemplar node. The fitness value $‘V_{f}’$ is computed using the expression below:

(16) $$V_{f}=\;\sum E_{r}+H_{c}+B_{p}+\frac{1}{D_{i,s}}$$

The expression above is composed of all computed parameters such as residual energy, hop count, distance and bandwidth. By means of computed fitness value, ACO further computes pheromone for each path for data transmission. The pheromone function ‘$P_{f}$’ is computed using the following expression:

(17) $$P_{f}=\;\frac{\tau_{i,j}{}^{\alpha }*V_{f}{}^{\beta }}{\sum_{i=0}^{n}\tau_{i,j}{}^{\alpha }*V_{f}{}^{\beta }}$$

where, $\tau_{i,j}$ indicates pheromone intensity, α and β are control parameters. The probability of selecting the path between the source CM and the exemplar node is expressed as follows:

(18) $$\rho_{k}=\;\frac{\;\alpha +\left[P_{f}\right]*\beta }{\sum_{i=0}^{n}\alpha +\left[P_{f}\right]*\beta }$$

The expression above indicates the probability of selecting a path as the transmission path between the source CM and the exemplar node.

(b) Revising rule

In this rule, the pheromone is updated in each iteration using the following expression,

(19) $$\tau_{i,j}\left(t+1\right)=\left(1-\varepsilon \right)\tau_{i,j}\left(t\right)+\;\varepsilon ∆\tau_{i,j}\left(t\right)$$

where ε represents local pheromone corrosion and $∆\tau_{i,j}\left(t\right)$ represents pheromone enhancement. By means of updating pheromone values in each iteration, ACO discovers multiple paths between the source CM and the exemplar node.

#### 3.3.2. FFA

From the selected multiple paths, FFA elects the best path between source CM and exemplar. FFA works on the principle of the flashing lights of fireflies. The reason behind selecting this algorithm for the best pathfinding is that it can generate an optimal result with a fast convergence rate and also provides better results with the aid of an attractiveness function. Here, the attractiveness degree of this algorithm is used to obtain the optimal solution in path selection between source and destination even under non-linear, high-density conditions. Hence, we select the firefly which has high attractiveness as the optimal path routing. Hence, we select this algorithm for optimal pathfinding in the WSN environment. Here, FFA computes the fitness function for succeeding metrics such as packet delivery ratio, expected delay and load.

(a) Packet Delivery Ratio

The packet delivery ratio is described as the ratio between the number of packets successfully transmitted by the source node and the number of packets successfully received by the exemplar node. It is expressed ‘${𝕕}_{r}$’ as follows:

(20) $${𝕕}_{r}=\frac{p_{s}}{p_{r}}$$

where $p_{s}$ represents packets successfully transmitted and $p_{r}$ indicates packets successfully received.

(b) Expected Delay

The expected delay is described as the time required to transmit data from the source to the destination node. This parameter is taken to measure the delay in the selected path, since it affects the energy consumption of the network. It is represented as ‘$E_{d}$’ that is expressed as follows:

(21) $$E_{d}=T\left(N_{CM_{i}},\;N_{e}\right)$$

where $T\left(N_{CM_{i}},\;N_{e}\right)$ represents the time required to transmit data between the source CM node $\;N_{CM_{i}}$ and the exemplar node $\;N_{e}$.

(c) Load

The load parameter is used to measure the load of each sensor node in the network. The load parameter increases automatically as energy consumption also increases. This parameter is expressed as ‘$N_{L}$’ and can be conveyed as follows:

(22) $$N_{L}=L\left(N_{i}\right)$$

where $L\left(N_{i}\right)$ represents the load of the node $N_{i}$.

FFA computes the fitness function for each node to find an effective path between the source CM and the exemplar node. The fitness function f(x) is expressed as follows:

(23) $$f\left(x\right)=\;\sum \frac{1}{E_{d}+N_{L}}+\;D_{r}\;$$

The light intensity of each node is computed using the above-explained fitness function  f(x). Light intensity ‘$I_{l}$’ is expressed as follows:

(24) $$I_{l}=\;I_{o\;}e^{-\gamma r}$$

where $I_{o\;}$ indicates the original light intensity and  γ represents the light absorption coefficient. The firefly that has the highest attractiveness function is selected as the optimal path between the source and destination. Here, attractiveness is represented as the brightness of the firefly which represents the path that is best among other paths between the source and destination. The attractiveness of FFA is computed using the expression below,

(25) $$\omega =\;\omega_{0}e^{-\gamma r^{2}}$$

where, $\omega_{0}$ indicates attractiveness at r = 0. Here, the firefly moves to the best position from i to j by using the below expression,

(26) $$N_{i}=N_{i}+\;\omega_{0}e^{-\gamma r^{2}}\left(N_{j}-N_{i}\right)+\;\delta \;\left(rand-\frac{1}{2}\right)\;$$

By using the above function, the firefly discovers the optimum path between the source CM and the exemplar node. With the aid of the proposed FFA, we find out the optimal path between the source and destination in the WSN environment. From the above process, our work effectually reduces network delay that in turn enhances network lifetime.

## 4. Performance Evaluation

In this section, we evaluate the performance of the E^2^S-DRL with existing methods using network lifetime, energy consumption, throughput and delay metrics. This section is further divided into three sections: simulation environment, performance metrics and comparative analysis. We compare our proposed work performances with existing methods.

### 4.1. Simulation Environment

Our proposed work is implemented in the Network Simulator 3 (NS3) tool, implemented in the Ubuntu operating system. NS3 is a discrete event simulator that provides simulations of different types of network; hence we preferred this simulator for proposed energy-efficient sleep scheduling in the WSN environment.

**Figure 3:**
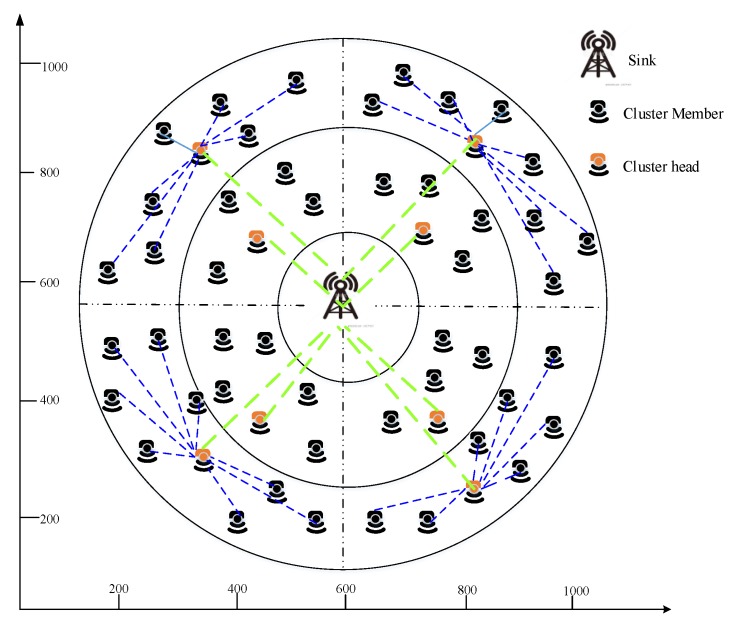
Simulation environment of our work.

Figure 3 illustrates the simulation environment of the proposed work. Our simulation environment is composed of 100 sensor nodes that are deployed in the three coronas. We position the sink node at the center of coronas; based on the sink node position each corona is further split into zones. Zones comprise clusters in which each cluster contains one cluster head respectively.

Table 2 illustrates the parameters used in our simulator. We position 100 sensor nodes in the 1000 * 1000 simulation area. The positioned sensor nodes have a 100 m communication range to transmit sensed information to neighbor nodes.

### 4.2. Performance Metrics

We evaluate the performance of our work using performance metrics such as network lifetime, energy consumption, throughput and delay that are summarized in the following sections.

#### 4.2.1. Network Lifetime

Network lifetime metric is used to measure the lifetime of the sensing network. Typically, network lifetime is referred to as the time at which the first node dies in the network. It is also defined as the operational time of the node during which it can be able to perform the allocated task. Network lifetime is represented as ‘${𝓃}_{\ell }’$ that can be conveyed as follows:

(27) $${𝓃}_{\ell }=\frac{{ℐ}_{ℰ}-{𝓌}_{𝓮}}{C_{p}+a_{𝓇}{ℛ}_{e}}$$

where ${ℐ}_{ℰ}$ denotes initial energy of the network, ${𝓌}_{𝓮}$ denotes wasted energy, $C_{p}$ denotes continuous power consumption of the network, $a_{𝓇}$ represents average sensor reporting rate and ${ℛ}_{e}$ represents estimated reporting energy.

#### 4.2.2. Energy Consumption

Energy consumption metric is described as the amount of energy consumed over sensing, data transmitting and data receiving in the network. It is represented as $E_{ℭ}$ that is represented as follows:

(28) $$E_{ℭ}=\;\sum D_{t}+\;D_{r}+\;{𝕤}_{f}$$

where $D_{t}$ represents the energy consumed during data transmission, $D_{r}$ represents the energy consumed during data receiving and ${𝕤}_{f}$ represents the energy consumed during sensing the field.

#### 4.2.3. Throughput

The throughput metric is described as the number of packets successfully received by the receiver in the given amount of time. It is represented as ‘T’ that is conveyed as follows:

(29) $$T=\;\frac{\sum_{i=1}^{n}p_{i}*p_{\ell }}{s\left(t\right)}$$

where $p_{i}$ represents the number of packets in node ‘i’, $p_{\ell }$ denotes data packet length and s(t) represents simulation time.

#### 4.2.4. Delay

Delay is defined as the time required to deliver sensed data from the source to the destination node. It is represented as ‘O’ that is conveyed as follows:

(30) $$O=\frac{T_{s}}{T_{d}}$$

where $T_{s}\;$ represents the time required to send data and $T_{d}$ represents data received by the destination node.

### 4.3. Comparative Analysis

In this section, we compare our proposed work’s performance with the existing methods LDC [43], iABC [27], cTDMA [35] and DRA [42] methods. The contributions of these methods are similar to the contribution of the E^2^S-DRL work in WSN. Hence, we select these methods to compare our proposed work. From this comparison, we show our proposed work is more efficient than the existing methods that are discussed above.

#### 4.3.1. Analysis of Network Lifetime

A network lifetime metric is a significant metric to analyze the performance of the proposed work. It demonstrates the efficiency of our proposed work in terms of the lifetime of the network. In our work, the network lifetime metric is measured by the number of nodes or simulation time.

**Figure 4:**
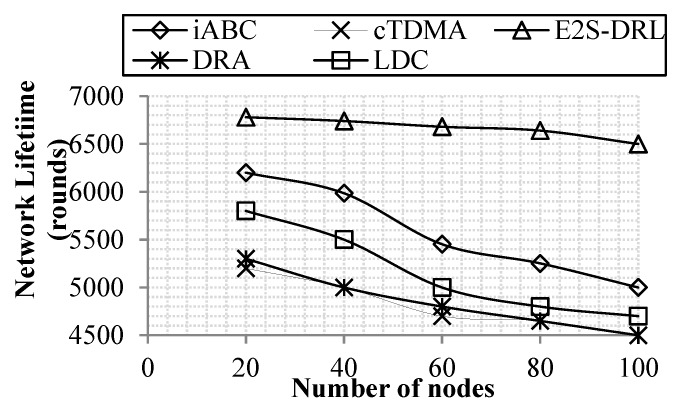
Comparison of network lifetime vs. the number of nodes.

Figure 4 shows that the proposed work achieves a high network lifetime compared to the existing methods DRA, LDC, iABC and cTDMA. The proposed duty cycling method achieves better network lifetime through reduce energy consumption, it provides sleep scheduling to the sensor nodes adaptively using the DRL algorithm. Furthermore, the ZbC method improves energy consumption via reduced energy consumption during data aggregation.

Our method achieves a maximum of 6780 rounds of network lifetime in presence of 100 nodes. Meanwhile, the existing methods, iABC and cTDMA, achieve the minimum network lifetime for 100 nodes. Here, the cTDMA and DRA methods have a minimum lifetime of 4600 rounds for 100 nodes compared to the other existing methods.

**Figure 5:**
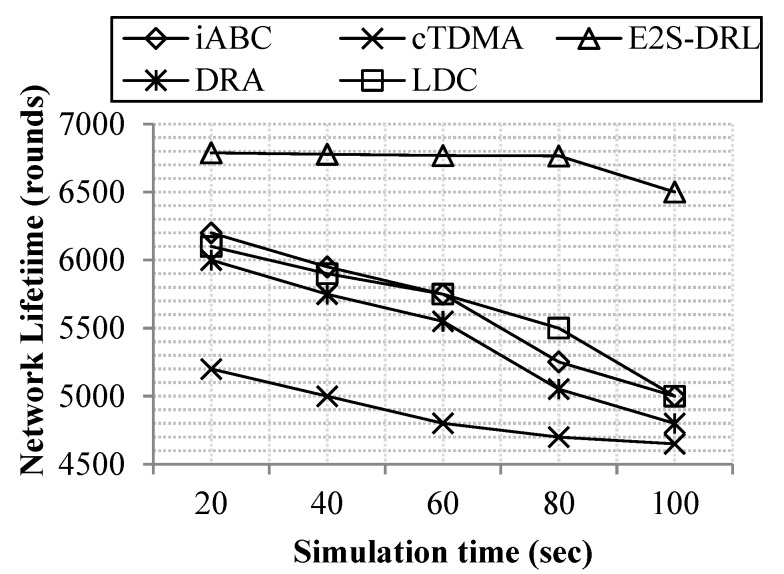
Comparison of network lifetime vs. simulation time.

Figure 5 illustrates network lifetime with respect to the simulation time that demonstrates our proposed work achieves better network lifetime compared to the existing methods DRA, LDC, iABC and cTDMA. The iABC method achieves the minimum network lifetime compared to the E^2^S-DRL, because the iABC method takes more objective functions to select the optimum cluster head and it also lacks the use of secondary information. The cTDMA method has a minimum network lifetime compared to the E^2^S-DRL method since it cannot change the scheduling slot dynamically, which reduces the energy of each sensor node drastically. Likewise, the LDC and DRA method has a low network lifetime compared to the E^2^S-DRL method due to the lack of concentration on the energy metric of the sensor nodes. These drawbacks pull down the network lifetime minimum for DRA, DRA, iABC and cTDMA methods. Network lifetime is decreased dramatically when simulation time increases. Our method achieves a maximum average network lifetime of 6720 rounds for 100 sec of simulation time whereas the existing methods DRA, LDC, iABC and cTDMA minimum average network lifetime is 5430 rounds, 5630 rounds, 5650 rounds and 4870 rounds for 100 sec of simulation time respectively.

#### 4.3.2. Analysis of Energy Consumption

Energy consumption is an important metric to improve the lifetime of the network. Since it is inversely proportional to the network lifetime, if the energy consumption of the network increases, then the lifetime of the network automatically decreases drastically. Thus, the energy consumption metric is reduced as much as possible in the network. In our network, the energy consumption of the network is measured with the number of nodes or simulation time.

**Figure 6:**
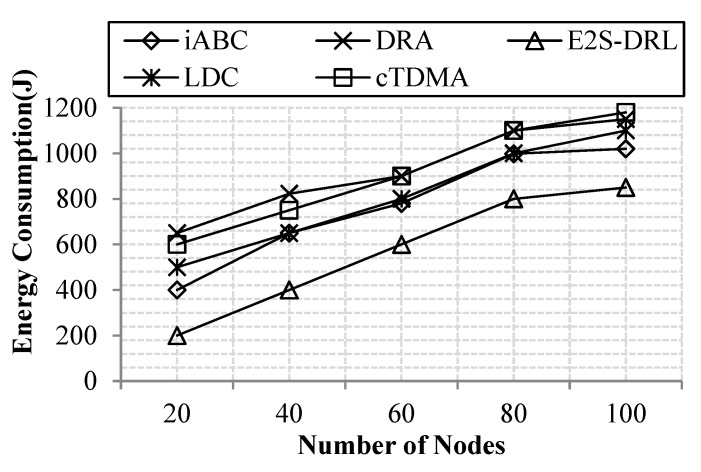
Comparison of energy consumption vs. the number of nodes.

Figure 6 illustrates the energy consumption with respect to the number of nodes, thus concluding that our work achieves better energy consumption compared to the existing methods like LDC, cTDMA, iABC and DRA. We deploy sensor nodes in the coronas field to support data aggregation efficiently, which reduces energy consumption. In addition, we furthermore reduce energy consumption through duty cycling and ZbC schemes. In duty cycling, the DRL algorithm is proposed, which provides scheduling slots for each sensor node based on the energy and states. The ZbC scheme reduces energy consumption through effective cluster head selection since the optimum cluster head can reduce energy consumption in data aggregation. These processes reduce the energy consumption of the network effectually. Our method achieves a minimum of 200 J and a maximum of 850 J for 100 nodes. The DRA method consumes more energy, it consumes a maximum of 1800 J for 100 nodes compared to the other existing methods.

**Figure 7:**
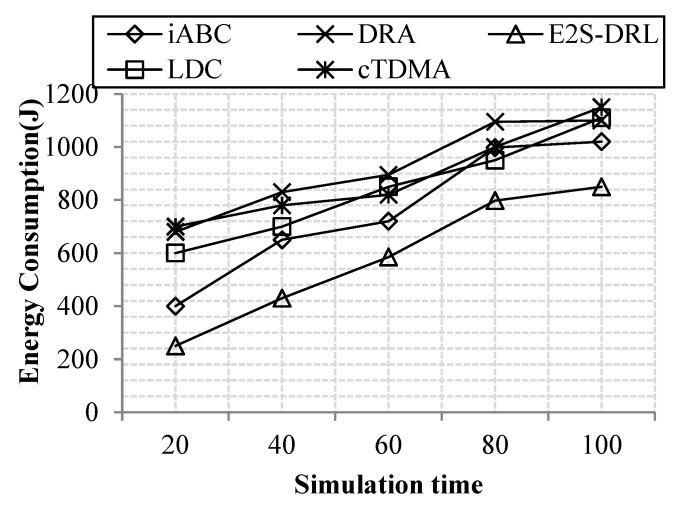
Comparison of energy consumption vs. simulation time.

Figure 7 demonstrates energy consumption with respect to the simulation time, thus concluding that our method achieves a reduced energy consumption compared to the simulation time. The DRA method has more energy consumption, since it does not elect an optimum cluster head to transmit sensed data. Meanwhile, the iABC method also consumes more energy due to its objective function evaluation. Likewise, LDC and cTDMA methods are achieved high energy consumption. The reason for this is that these methods do not concentrate on proper slot allocation for the sleep and wakeup periods of the sensor nodes. These drawbacks lead to more energy consumption of DRA and iABC methods. Our proposed method achieves the average energy consumption of 583 J for 100 s of simulation time. Whereas, LDC, cTDMA, DRA and iABC methods consume average energy of 842J, 890J, 920 J and 757 J for 100 s of simulation time respectively. From the comparative results, we conclude that our work achieves better energy consumption compared to the existing cTDMA, LDC iABC and DRA methods.

#### 4.3.3. Analysis of Throughput

The throughput metric is related to the performance of the proposed network. Hence, throughput is increased as much as possible in our work. In performance evaluation, the throughput metric is measured using the number of nodes or the number of rounds.

**Figure 8:**
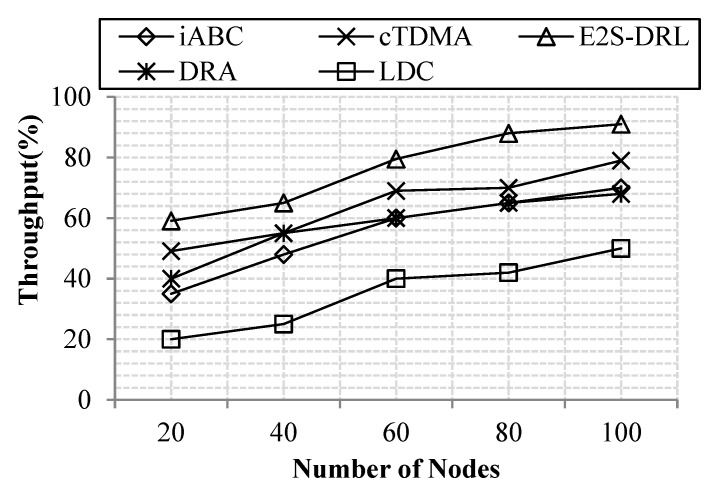
Comparison of throughput.

Figure 8 describes how our proposed work produces high throughput compared to the existing methods such as DRA, LDC, iABC and cTDMA. In existing methods like iABC and cTDMA loose data packets are in transmission due to poor path selection between the source and the sink node. Likewise, DRA and LDC methods do not concentrate on the buffer-related and link-related metrics to route the data packet, thus reducing the throughput of the WSN effectually. Hence, we propose an effective routing method that efficiently reduces data packet loss through minimizing delay in the network. The delay of the network is reduced through an effective selection of paths between source and destination. Here, we pursue ACO and FFA to select an optimum path between the source and destination. The ACO selects multiple paths between source and destination, from which FFA elects the best path between the source and destination. This way of selecting a path diminishes delay effectually that in turn improves the throughput of the proposed work. Our work achieves a maximum of 91% throughput for 100 nodes. Meanwhile, DRA achieves a maximum of 68%, LDC achieves a maximum of 50%, iABC achieves a maximum of 70% and cTDMA achieves a maximum of 79% for 100 nodes. Thus, our results show that our proposed work achieves better throughput that in turn improves the performance of the network.

#### 4.3.4. Analysis of Delay

The delay metric is significant for improving the lifetime of the network since it is directly proportional to the energy consumption and network lifetime metric. Thus, our work reduces delay as much as possible. Usually, the delay metric is measured using the number of nodes or the number of rounds.

**Figure 9:**
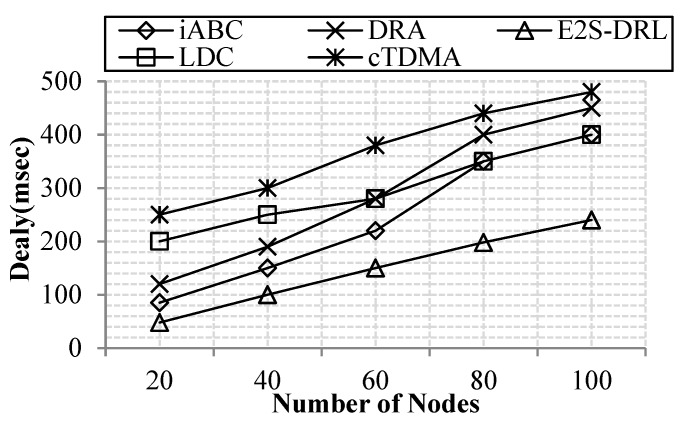
Comparison of delay.

Figure 9 demonstrates that the delay of proposed work is minimal compared to the existing methods LDC, cTDMA, iABC and DRA. If the number of nodes increases, the automatic delay also increases. The above comparison clearly shows that our work transmits packets with minimum delay. Our scheduling scheme reduces delay by allocating slots based on the packets in its buffer. Furthermore, the delay is reduced through effective path selection between source and destination. Here, ACO selects multiple paths between source and destination and FFA selects the best path among multiple paths that in turn reduces delay effectively. Our method achieves a minimum of 240 ms for 100 nodes. The cTDMA and DRA methods have poor data transmission, since it has a maximum delay of 450 and 480 ms for 100 nodes, respectively, due to the lack of consideration of distance- and buffer-related metrics for comparison. Likewise, iABC and LDC methods also acquire high delay as 400 ms for 100 nodes. It is due to the slow processing performance of the utilized algorithms. From the above comparison, we conclude that our method achieves better data transmission with minimum delay in the network.

#### 4.3.5. Memory Consumption Analysis

In this section, we analyze the performance of the proposed work with memory consumption. Here, we have also compared the memory consumption of the proposed work with the existing methods including iABC, LDC, DRA and cTDMA. It is measured with the aid of varying the number of nodes in the network.

**Figure 10:**
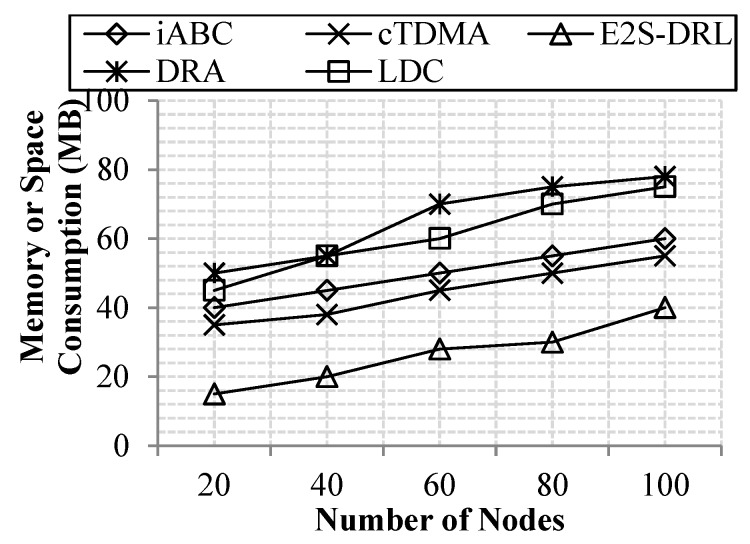
Comparison of memory or space analysis.

As represented in Figure 10, our E^2^S-DRL achieves better results in memory or space consumption compared to the other existing methods. This is achieved because of our proposed algorithms for clustering, duty cycling and routing. Our proposed algorithms, including PSO-AP, DRL and ACO-FFA, are consuming less memory to select the optimal cluster head, mode of operation and optimal path. Our routing selects the path with high energy and minimum delay to transmit data to the destination. Hence this reduces the memory consumption of the sensor node effectually. Meanwhile, the existing methods acquire high memory consumption due to its poor processing performance of the selected algorithms. It leads to high memory consumption in each sensor node in the network. Our method reduces 40% in memory consumption compared to the existing methods including iABC, DRA, cTDMA and LDC.

#### 4.3.6. Computational Complexity Analysis

This portion discusses the performance of the proposed work in terms of complexity. Here, we compare the time complexity of the proposed optimization algorithm and reinforcement algorithm with the existing iABC and cTDMA algorithms.

From Table 3, we proved that the computation complexity of the ES-DRL is less than the other existing methods present in the clustering, duty cycling and routing. In clustering, the existing iABC has O(2*P/2*D*I^2^) where P represents the number of population, D represents the dimension and I represents the number of iteration. Meanwhile, our E^2^S-DRL has less complexity as O(P*I+n^2^). For duty cycling, cTDMA has a time complexity of three times the number of operations to be performed i.e., 3O(n). Here, the n represents the number of operations. Our proposed E^2^S-DRL has only O(n) operations to perform duty cycling. Likewise, LEACH routing has the nO(L) operations to complete the routing in WSN where our proposed E^2^S-DRL only consumes O(n^2^+nlogn) time to complete the routing. From the above analysis, we conclude that our proposed E^2^S-DRL achieves less computational complexity compared to the existing algorithms.

## 5. Discussion

This portion discusses the highlights of the E^2^S-DRL work in terms of improving the network lifetime and network delay. To attain this, our E^2^S-DRL proposed three phases: clustering, duty cycling and routing. The advantages of proposing these phases are listed as follows:

- *Clustering:* In this phase, we mitigate the energy consumption that occurred during the aggregation of the data in the cluster head node. In cluster-based WSN, energy consumption during data aggregation is a big issue that leads to frequent cluster head election. These problems are resolved in our proposed clustering by selecting an optimal cluster head for data aggregation based on the effective parameters.
- *Duty Cycling:* This phase reduces the energy consumption of the individual sensor node by exploiting the DRL algorithm. Using this algorithm, each sensor node in the network decides its mode of operation perfectly. Using this, the proposed method reduces the energy consumption of the individual node effectively.
- *Routing:* The network delay is one of the significant issues in the WSN; it reduces the performance of the proposed system. To attain less network delay, our E^2^S-DRL method performs the multiple paths based on optimal path selection in WSN. It initially selects the multiple paths between source and destination node. From the selected multiple paths, our E^2^S-DRL chooses a better path to transmit the sensed data packet to the destination. This reduces the delay incurred during the data transmission between the source and sink node.

The algorithms incorporated in the aforesaid phases are discussed with their benefits in terms of the performance metrics in Table 4.

The performance of the proposed work is compared with the performance of the existing methods including iABC, cTDMA, DRA, MPACO and LDC with the average results acquired for the following metrics: network lifetime, energy consumption, throughput and delay. The numerical results obtained from the E^2^S-DRL and existing methods are deliberated in the Table 5. From this comparison, it is perceived that our proposed E^2^S-DRL algorithm attains better performance compared to the proposed methods. Here, #N represents the number of nodes and S.T represents the simulation time.

## 6. Conclusions and Future Work

To date, reducing the energy consumption and network delay in WSN is essential due to its evolving applications. To improve network lifetime and reduce the network delay, we propose energy-efficient sleep scheduling using the DRL algorithm (E^2^S-DRL). Our proposed method comprises three major phases, clustering, duty cycling and routing. In the first phase, the clustering operation is functioned via the ZbC scheme that is executed through a hybrid PSO and AP algorithm that reduces energy consumption during data aggregation. Here, PSO is used to select the optimal exemplar to form the AP clusters. In the duty cycling phase, the DRL algorithm is proposed that effectively schedules each node based on the energy and state adaptively that in turn improves network lifetime via reduced energy consumption and also reduces data collision among sensor nodes. The routing is adopted to reduce the network delay that is executed by employing ACO and FFA. Here, the ACO selects the multiple paths between source and destination node. From the multiple paths, the FFA selects the optimal path to transmit the packet to the sink node. At last, we evaluate our proposed work performance with existing methods like LDC, iABC, cTDMA and DRA using network lifetime, throughput, energy consumption and delay metrics. It is concluded that our method reduces 40% in delay and energy consumption and increases 35% in network lifetime and throughput compared to the existing methods.

In the future, we intend to propose mobile sink-based data aggregation in WSN in order to improve the performance of the sensor nodes. Besides, we also concentrate on application scenario-based research in WSN i.e., forest fire monitoring and air pollution monitoring.

## Figures and Tables

**Table 1:** Description and demerit of the related works.

Author Name	Method	Contribution	Objective	Demerits
Palvinder et al. [27]	iABC	It selects the optimal cluster head for data transmission in WSN.	To select the cluster head fast using the optimization algorithm.	The iABC-based cluster head selection lacks in analyzing the secondary information of the sensor nodes. This results in frequent cluster head selection.
Trong et al. [28]	BAT	It selects a cluster head and forms clusters in the sensor network.	To select the optimal cluster head to reduce energy consumption.	It consumes more time to select the cluster head. This reduces the energy consumption.
Islam et al. [29]	LEACH	It forms clusters and selects a cluster head using an optimization algorithm.	To perform efficient data aggregation in WSN.	The cluster head selection is not optimal that affects the data aggregation efficiency.
Jaeyoung et al. [30]	E-LEACH	It forms clustering and utilizes the distributed algorithm to pass the message.	To form clusters effectually to reduce energy consumption.	More parameters are required to select the best cluster head. Hence, it has frequent clustering in the network.
Kozlowsi et al. [31]	EEDC	It provides a duty-cycle based on transmitting and receiving energy.	To provide an energy-efficient duty-cycle.	It increases the delay during data transmission that tends to packet loss.
Mohamed et al. [35]	cTDMA	It allocates slots to transmit data packets based on the cluster head occurrence count.	To increase the lifetime of the WSN network.	It cannot change the transmission slots dynamically that leads to more energy drainage.
Ngoc et al. [36]	ISS	It provides a slot to each node based on the number of data packet count.	To minimize the data transmission delay in WSN.	It has high energy consumption due to a lack of consideration of energy-oriented metrics.
Mohammed et al. [37]	Modified TDMA	It allocates slots using set and steady phases.	To reduce the energy consumption of the cluster head.	It follows a complex computation process that decreases the performance of the system.
Supreet et al. [38]	ACO-PSO	The cluster-based routing is performed using the hybrid ACO-PSO algorithm.	To provide better data aggregation in data transmission.	The distance parameter only considered for next-hop selection thus increases the energy consumption.
Vishal et al. [39]	ACO	It routes the packet to the optimal path.	To balance the energy among the cluster head node in WSN.	The distance and energy are not sufficient to attain less delay in WSN routing.
Vishal et al. [40]	MPACO	It routes the packet by selecting the optimal path.	To design energy-efficient routing in WSN.	The parameters considered for routing are not sufficient to reduce packet loss in WSN.
Hana et al. [41]	MH-GEER	It provides cluster-based routing to route the sensed data.	Reducing the sensor node energy consumption to increase performance.	The next-hop is selected based on the energy, which thus leads to an increase in the load of each sensor node.
Feng et al. [42]	DRA	It selects the optimal path to transmits the data packet.	To prolong the lifetime of the WSN.	The distance between the source and destination node is not considered thus increases latency.
Jiang et al. [43]	LDC	It provides Low-Latency and Energy-Efficient Data Preservation Mechanism	To reduce the latency and energy consumption in WSN	The nodes are woken up based on the neighbor’s wakeup time which leads to the increase in energy dissipation of nodes
Vijayalakshmi and Manickam [44]	SDMA	It provides data gathering in WSN using SDMA and PSO	To prolong the lifetime of the WSN	CH selection was not effective due to the absence of significant parameter consideration such as distance and energy.

**Table 2:** Simulation parameter.

Parameters		Value
**Network Parameters**	Simulation Area	1000 * 1000 m
	Number of sensor nodes	100
	Number of Sink node	1
	Initial Energy of the Node	1200 J
**Packet Parameters**	Number of Packets	≈1000
	Number of retransmission	Max 7
	Packet size	512 KB
	Packet Interval	0.1 s
	Traffic Type of Packet	Constant Bit Rate (CBR)
**Communication Parameters**	Sensor Communication Range	100 m
	Data Rate	20 Mbps
**Transmission Slot Parameters**	Number of Slots	16
	Data Packet length	840 bits
	Slot length	1050 bits
	Slot Duration	10 μs
**PSO Parameters**	Number of Particles	[20–80]
	Maximum Inertia Weight	0.9
	Minimum Inertia Weight	0.1
**ACO Parameters**	Number of Ants	100
	α	0.6
	β	0.6
**FFA parameters**	Firefly population	100
	ω	0.9
	γ	1
Number of Run		1000
Simulation time		100 s

**Table 3:** Time complexity analysis.

Process	Method	Time Complexity
Clustering	iABC	O(2*P/2*D*I^2^)
	PSO-AP	O(P*I+n^2^)
Duty Cycling	cTDMA	3O(n)
	DRL	O(n)
Routing	LEACH	nO(L)
	ACO-FFA	O(n^2^+nlogn)

**Table 4:** Benefits of the proposed algorithms.

Proposed Algorithm	Benefits Related to Performance Metrics
PSO-AP	The PSO selects the optimal exemplar for the AP algorithm based on the energy-related metrics. Hence, the selected cluster head has the ability to sustain for a long time. This reduces the energy drain of the cluster head effectively. Therefore, the E^2^S-DRL method enhances energy consumption in WSN. Besides, the proposed AP algorithm forms clusters quickly compared to the traditional clustering algorithms such as K-means, etc. Thus, this reduces the time required during the setup phase.
DRL	The proposed DRL algorithm provides the proper mode of operation for each sensor node. For this purpose, it considers the buffer size parameter of each sensor node. Based on the buffer size, it allocates the modes to each node. This results in the reduction of energy consumption which tends to increase in the network lifetime drastically. Besides, it also reduces the network delay by considering the buffer size-based mode of operation allocation.
ACO-FFA	The throughput of the proposed work is increased by selecting an optimal path from the multiple paths. Here, ACO selects the best multiple paths for transmission with less amount of time. From the selected multiple paths, FFA selects the optimal path to transmit the message between the source and sink node. Here, expected delay, PDR and load parameters are considered to achieve less delay during packet transmission. These metrics also increase the throughput of the proposed system.

**Table 5:** Numerical results of proposed work.

*Methods*	*Performance Metrics*					
	*Network Lifetime (rounds)*		*Energy Consumption (J)*		*Throughput (%)*	*Delay (ms)*
	*#.N*	*S.T*	*#.N*	*S.T*		
*iABC*	5577	5630	769	757	55	241
*DRA*	4850	5430	924	920	57	288
*LDC*	5160	5650	810	842	35	296
*cTDMA*	4810	4870	906	890	64	370
*MPACO*	1200	1150	300	280	60	365
***E^2^S-DRL***	***6668***	***6719***	***570***	***582***	***90***	***147***
